# Suicidality in patients with bipolar depression: findings from a lower middle-income country

**DOI:** 10.1192/bjo.2021.207

**Published:** 2021-06-18

**Authors:** Siqi Xue, John Hodsoll, Ameer Bukhsh Khoso, Muhammad Omair Husain, Imran B Chaudhry, Allan H Young, Juveria Zaheer, Nusrat Husain, Benoit Mulsant

**Affiliations:** 1 University of Toronto; 2 king's college london; 3 Pakistan Institution of Living and Learning; 4 Centre for Addiction and Mental Health; 5 University of Manchester

## Abstract

**Aims:**

Among low- and middle-income countries (LMICs), bipolar disorder is recognized as one of the leading causes of disease burden for adults and is associated with marked suicide risk. There are limited data on suicidal ideation in bipolar disorder from LMICs. This study presents cross-sectional data on the prevalence of suicidality and associated patient characteristics among patients with bipolar depression in Pakistan, a lower-middle income country and the fifth most populous country in the world.

**Method:**

Participants were recruited through outpatient psychiatric clinics in between 2016–2019 in Karachi, Lahore, Hyderabad and Rawalpindi between 2016–2019. Participants were aged 18 to 65 years with a known diagnosis of bipolar disorder and currently in a depressive episode. Suicidality was assessed using the suicide item of the 17-item Hamilton Depression Rating Scale (HAM-D) and levels of severity were categorized as absent, mild/moderate, or severe. Biometric data and biomarkers were obtained. Descriptive statistics were used to describe prevalence and proportional odds regression models were applied to establish correlates to suicidal ideation.

**Result:**

Among the 266 participants, 67% indicated suicidality of any level and 16% endorsed severe suicidality. Lower body mass index (BMI) (OR = 0.93, 95% CI = 0.88–0.98), higher HAM-D score (OR = 1.29, 95% CI = 1.16–1.43), lower C-reactive protein (CRP) level (OR = 0.53, 95% CI = 0.40–0.70), and increased number of inpatient hospitalizations (OR = 1.16, 95% CI = 1.03–1.31) were identified as significant predictors of suicidality in the fully adjusted regression model. No patient demographic data, including age, gender, marital status, socioeconomic status, and years of education were associated with severity of suicidality.

**Conclusion:**

There exists a high prevalence of suicidal ideation among patients with bipolar depression in Pakistan. Our findings add to the limited literature on suicidality in bipolar disorder in the LMIC context and suggest roles of biological variables such as BMI and CRP level in predicting suicidal ideation and potentially suicidal behaviours in bipolar depression. More studies are needed to see whether such findings can be replicated in other similar LMIC settings, and to explore potential physiological pathways linking BMI, inflammatory biomarkers and suicidality in bipolar disorder.

